# Role of automated detection of respiratory related heart rate changes in the diagnosis of sleep disordered breathing

**DOI:** 10.3389/frsle.2023.1162652

**Published:** 2023-06-22

**Authors:** Scott Maresh, Adhithi Keerthana Athikumar, Nabila Ahmed, Shivapriya Chandu, Joel L. Prowting, Layth Tumah, Abed A. Najjar, Hamza Khan, Muna Sankari, Oluwatobi Lasisi, Laurel A. Ravelo, Paul E. Peppard, M. Safwan Badr, Abdulghani Sankari

**Affiliations:** ^1^Sleep Research Laboratory, John D. Dingell Veterans Affairs Medical Center, Detroit, MI, United States; ^2^Department of Internal Medicine, Wayne State University-School of Medicine, Detroit, MI, United States; ^3^Department of Kinesiology and Health Science, York University, Toronto, ON, Canada; ^4^Population Health Sciences, University of Wisconsin-Madison, Madison, WI, United States; ^5^Department of Medical Education, Ascension Providence Hospital, Southfield, MI, United States

**Keywords:** sleep, heart rate, R-R interval (RRI), pulse ox meter, ECG, polysomnography, sleep apnea and cardiovascular disease

## Abstract

**Study objectives:**

The objective of this study was to determine whether electrocardiogram (ECG) and heart rate accelerations that occur in the vicinity of respiratory events could predict the severity of sleep-disordered breathing (SDB).

**Methods:**

De-identified polysomnogram (NPSG) recordings from 2091 eligible participants in the Sleep Heart Health Study (SHHS) were evaluated after developing and validating an automated algorithm using an initial set of recordings from 1,438 participants to detect RR interval (RRI) dips in ECG and heart rate accelerations from pulse rate signal. Within-subject comparisons were made between the apnea-hypopnea index (AHI) and both the total RRI dip index (total RRDI) and total heart rate acceleration index (total HRAI).

**Results:**

The estimated AHIs using respiratory-related HRAI correlated with NPSG AHI both in the unadjusted and adjusted model (B: 0.83 and 0.81, respectively *P* < 0.05). Respiratory-related HRAI had a strong agreement with NPSG AHI (intraclass correlation coefficient-ICC: 0.64, whereas respiratory-related RRDI displayed weaker agreement and ICC: 0.38). Further assessment of respiratory-related HRAI (≥5 events/h) showed a strong diagnostic ability (78, 87, 81, and 56% agreement for traditional AHI cutoffs 5, 10, 15, and 30 events/h, respectively). At the AHI cutoff of 5 events/h the receiver operating curves (ROC) revealed an area under the curve (AUCs) of 0.90 and 0.96 for RE RRDI and RE HRAI respectively.

**Conclusion:**

The automated respiratory-related heart rate measurements derived from pulse rate provide an accurate method to detect the presence of SDB. Therefore, the ability of mathematical models to accurately detect respiratory-related heart rate changes from pulse rate may enable an additional method to diagnose SDB.

## Introduction

Sleep-disordered breathing (SDB) is a deleterious medical condition characterized by episodic asphyxia and interruption of normal sleep (Hosselet et al., 2001) and is associated with significant morbidity such as cardiovascular disease (Bauters et al., 2016). The severity of an individual's SDB is determined by calculating their apnea-hypopnea index (AHI), which is the total number of apnea and hypopnea events experienced per hour of sleep. This metric is highly variable and dependent on the type of sleep test performed as well as the expertise of the individual scoring the sleep study (Berry et al., 2017). Due to the high cost and specialized equipment required to perform a full nocturnal polysomnography (NPSG) sleep test, less costly procedures with wider access and applicability have been developed (Kapoor and Greenough, 2015). One such example is type 3 home sleep apnea tests, which have become the default method of diagnosis for many patients (Bozkurt et al., 2019). Despite the usefulness of these tests, they may underestimate the severity of the disease or miss the diagnosis in patients at high risk of SDB due to a lack of electroencephalogram (EEG) which allows the detection of arousals (Punjabi et al., 2013).

Advancements in technology and computing power now allow for the formulation of algorithms that can analyze large amounts of data with a high level of accuracy in an automated way. A recent study demonstrated that a neural network-based analysis of the oxygen saturation signal could provide an accurate assessment of obstructive sleep apnea (OSA) severity in snoring children (Hornero et al., 2017). Another potentially useful physiological measure for estimating SDB is assessing fluctuations in heart rate. In previous studies in individuals with sleep apnea, changes in electrocardiogram (ECG) signal modeling was utilized to detect with high accuracy the presence of apnea (Radha et al., 2019; Iwasaki et al., 2021). A recent prospective community cohort study examined the relationship between nocturnal heart rate changes (via single lead ECG) and cardiovascular outcomes over a 15-year period and found that an elevated frequency of nocturnal shortened RR intervals (RRI) was associated with an increased risk of cardiac-related events and mortality later in life (Sankari et al., 2019). Considering that heart rate is non-invasive and can be easily obtained from both sleep study recordings as well as wearable devices, developing techniques to interpret its relationship to SDB should be prioritized. Previous studies assessed the role of detecting heart rate changes in the diagnosis of prognosis of SDB however these studies either used ECG signals which are limited to full polysomnography studies, were in a pediatric population (Hornero et al., 2017) or used small sample sizes, or used heart rate variability algorithms (Shao et al., 2019). At present, it is unknown whether an automated analysis of the heart rate signal from readily available signals such as pulse rate from pulse oximeter and/or ECG can be used to estimate SDB in adults (Mejía-Mejía et al., 2020). If a strong association is found, it may allow for a more accurate diagnosis in limited home sleep apnea attests and assist in the prediction of the likelihood of developing cardiovascular disease (CVD) outcomes in an adult population (Azarbarzin et al., 2021).

Our laboratory has developed an algorithm that can measure respiratory-related heart rate changes that occur during sleep studies (Sankari et al., 2019). The objective of this study was to examine whether our novel algorithm could use heart rate accelerations derived from a pulse oximeter and single-lead ECG to detect SDB using data collected from the Sleep Heart Health Study (SHHS). We hypothesized that the nocturnal heart rate changes obtained from the pulse signal and ECG would provide an accurate method to detect the presence of SDB.

## Methods

### Participants

We studied individuals from the SHHS database accessed from the National Sleep Research Resource (NSRR) (Quan et al., 1997; Zhang et al., 2018). The protocol was approved by the Human Investigation Committee of Wayne State University.

### Cohort description

The SHHS was comprised of 5804 adults aged 40 and older (at the cohort's inception) that completed unattended at-home NPSG between the dates of November 1^st^, 1995, and January 31^st^, 1998, and were tracked for cardiovascular disease outcomes until April 1^st^ of 2006. SHHS participants were eligible to be included in this study if they had complete NPSG data, including ECG and pulse oximeter recordings, had no prior CVD event, and did not use beta blockers on the night of the sleep study or at any other point during follow-up.

### Predictor

**Figure 1:**
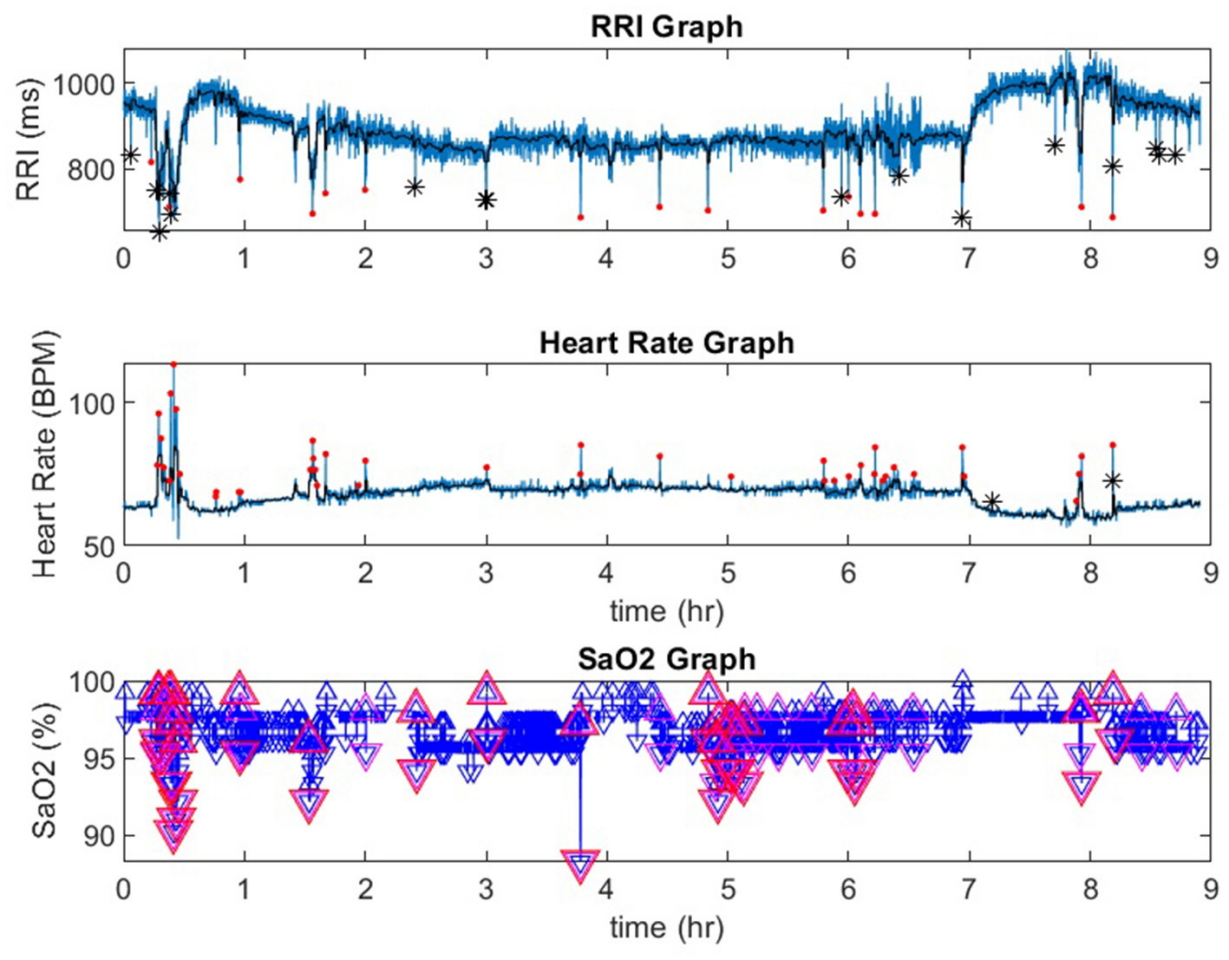
A representative record from a participant in the Sleep Heart Health Study. In the RRI channel, red dots indicate RRI dips and black stars indicate artifacts. In the heart rate channel red dots indicate heart rate accelerations. In the SaO_2_ channel, the red triangles indicate desaturations that are 3% or greater. RRI, R-R interval; SaO_2_, pulse oximetry. ^*^indicate artifcats.

**Figure 2:**
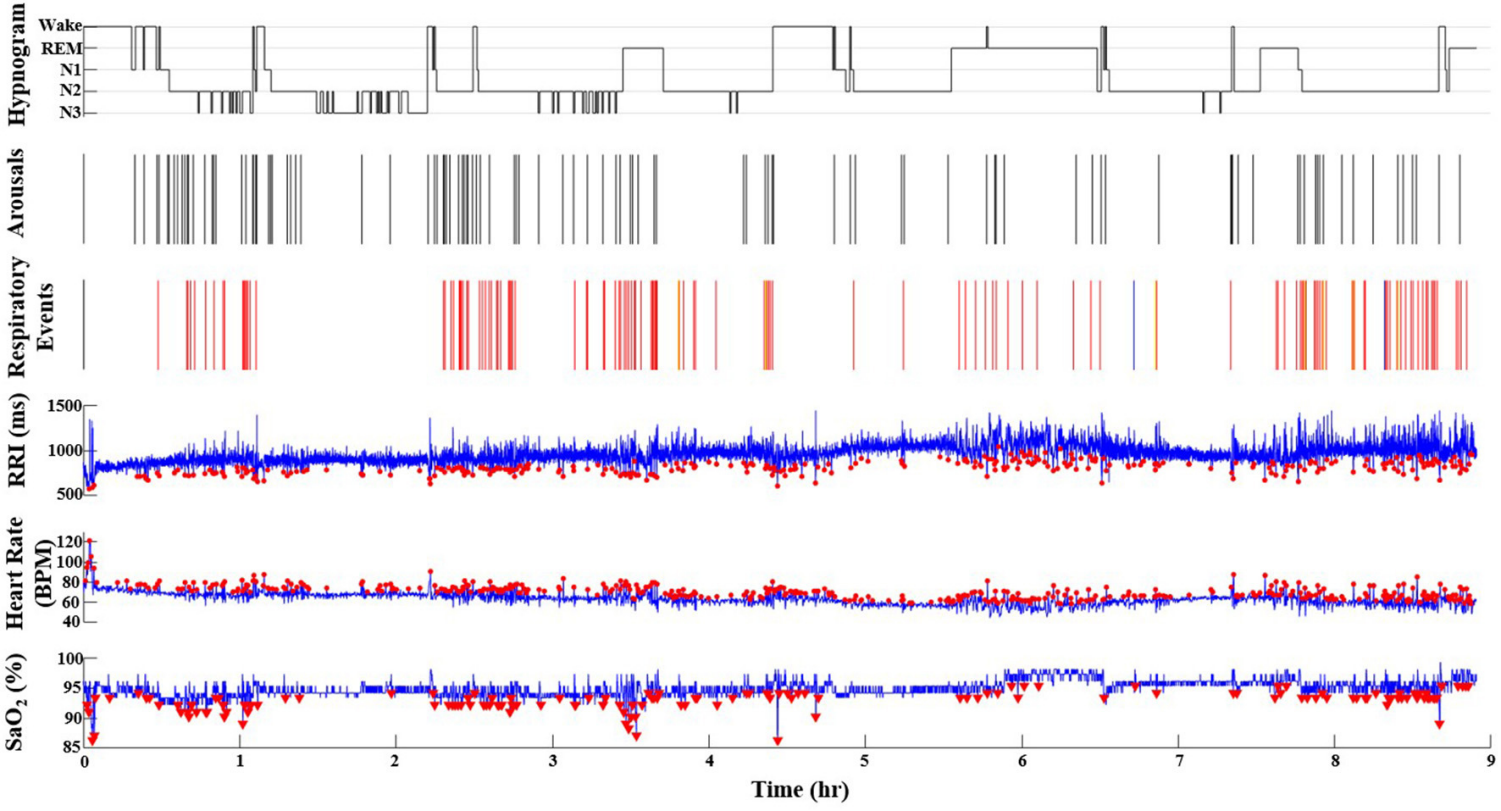
A representative record from a participant in the Sleep Heart Health Study who has SDB (AHI = 16.9 events/h). The red dots represent the O_2_ desaturations (ODI = 11.5), heart rate accelerations (HRAI from pulse oximeter), and RRI dips (from ECG) throughout the duration of the PSG recording (9 h). Note the incremental increase of values from ODI, AHI to HRAI, and RRI dips. In the respiratory events channel, red lines indicate hypopneas, blue lines indicate obstructive apneas and yellow lines indicate central apneas. In the hypnogram, the wake-sleep stages are indicated as wake, N1, N2, N3, and REM. In the RRI channel, red dots indicate RRI dips. In the Heart Rate channel, red dots indicate heart rate accelerations. In the SaO_2_ channel, the red triangles indicate desaturations that are 3% or greater. N1, non-REM stage N1; N2, non-REM stage N2; N3, non-REM stage N3; RRI, R-R interval; SaO_2_, pulse oximetry.

The main predictor variables were the hourly rate of heart rate intervals (RRI) (defined as the time interval between a successive pair of QRS complexes) and the hourly rate of heart rate (HR) accelerations (HRA) over an entire NPSG (as illustrated in the supplements). RRI was measured from the ECG signal sampled at 125 Hz by using software that could detect R waves in LabChart with a heart rate variability module (AD Instruments, Colorado Springs, Colorado, USA) (Figure 1). Individuals performing data analysis were blinded to the SDB severity of the participant. Each tachogram created by LabChart was first closely examined for artifacts and assessment of upper and lower limits for RRI values to exclude non-physiological beats. The ECG signal was then retrieved to a MATLAB R2019b program (MathWorks, Natick, Massachusetts, USA) developed and validated by our group to obtain an RRI signal for the entire night as depicted in Figures 1, 2 and Supplementary Figures S1–S5) (Sankari et al., 2019). The RRI signal is exported from LabChart and divided into 1-min segments. The program calculated the average RRI value for each given segment, and considered it to be the baseline, then compared each individual RRI value to that average as a baseline. The program marked all RRI values that were < 90% of the average RRI value in the 1-min segment to which it belongs and grouped them together. If two of these groups of consecutive beats were < 10 seconds apart, they were merged. In each group of these RRI points, the one with the lowest RRI value was an RRI dip (red dot in Figure 1). For these beats to be considered they must have at least two consecutive values that were less than the threshold (90%) of the baseline to be considered as a dip; otherwise, they were considered to be noise that was marked and not analyzed (more details are available in the Supplementary material). The 90% threshold was selected because previous work demonstrated that it correlated with most respiratory events (Sankari et al., 2017). The total number of dips was calculated and divided by the total recording time of the NPSG to determine the total index of RRI dips (RRDI). For the calculation of respiratory-related RRDI (RE RRDI), after the RRI dips were selected, the program linked the RRI dips to the specific respiratory events automatically if they occurred during or up to 30 seconds after the end of a respiratory event. The scoring of respiratory events is not automated given that they were scored in the SHHS files. The RE RRDI was calculated as the total number of respiratory-related RRI dips divided by the total NPSG recording time in hours.

Heart rate accelerations (HRA) were assessed by using the pulse rate signal sampled at 1 Hz in the NPSG recording from SHHS. Heart rate values were converted to heart rate intervals mathematically and analyzed in the same way as above. Using correlation analysis at different thresholds we found that the 5% threshold of HRA provided the highest correlation to AHI from NPSG. Therefore, the HRA index (HRAI) was calculated by dividing the total number of HRAs more than 5% from the baseline HR for the 1-min time segment in which they occurred by the total recorded time of the entire NPSG. The total HRAI and respiratory-related HRAI were calculated in the same fashion as described above for RRDI.

### Sleep study analysis

Participants in the SHHS study underwent overnight 12-channel EEG-based NPSG (Compumedics Sleep Watch; Abbotsford, Victoria, Australia) at home using a standard protocol. Channels included oximetry, heart rate, chest wall movement, abdominal wall movement, nasal/oral airflow, central electroencephalogram (EEG) (bilateral), electrooculogram (bilateral), chin electromyogram, and ECG. An apnea was defined as a complete or almost complete cessation of the airflow that lasted for at least 10 seconds. Hypopneas were scored using the American Academic of Sleep Medicine (AASM) 2012 criteria (Berry et al., 2017) (defined as reductions in airflow or chest wall movement to below approximately 70% of the baseline for at least 10 seconds and accompanied by arousal or a blood oxygen desaturation of 3% or more). The AHI was calculated by summing the number of apneas and hypopneas and dividing by the total sleep time in hours (from light on to light out).

### Statistical analysis

Total RRDI and HRAI and respiratory-related RRDI and HRAI were compared to AHI. A Bland-Altman plot and intraclass correlation coefficient (ICC) were used to directly assess the agreement between the AHI and the respiratory-related HRAI and RRDI. For ICC, the following cut-offs were used to interpret the scores: < 0.50 = poor, 0.50 to 0.75 = moderate, 0.75 to 0.90 = good, and >0.90 = excellent (Koo and Li, 2016). Diagnostic testing was done to assess how well these metrics could predict SDB with different AHI cutoffs (AHI ≥ 5 events/hr, AHI ≥ 10 events/hr, AHI ≥ 15 events/hr, AHI ≥ 30 events/hr) for RRDI ≥ 5, RE RRDI ≥ 5, HRAI ≥ 5, RE HRAI ≥ 5. Sensitivity, specificity, positive predictive value (PPV), negative predictive value (NPV), and agreement were calculated. A Spearman rank correlation was used to assess the monotonic relationship and a Pearson product-moment correlation was used to assess linear relationships between several different variables, with the following cutoffs used to interpret results: 0.00–0.10 = negligible correlation, 0.10 to 0.39 = weak correlation, 0.40 to 0.69 = moderate correlation, 0.70 to 0.89 = strong correlation and 0.90–1.0 = very strong correlation (Schober et al., 2018). To visually examine how well total RRDI/HRAI and RE RRDI/HRAI can predict AHI severity at various cut points, we created receiver operating curves (ROC). We computed ROC curves for 4 severity levels of AHI (5, 10, 15, and 30 events/h). The higher the Area Under the ROC Curve (AUC), the better the model (i.e., RE-RRDI) is at predicting 0 cases as 0 and 1 cases as 1. When AUC is 0.7, it means there is a 70% chance that the model will be able to distinguish between positive cases and negative cases. The ROC curves and AUC were calculated using Proc Logistics with the ROC option in SAS. We also examined correlations between AHI and total RRDI/HRAI and RE RRDI/HRAI. We first examined raw correlations and then we calculated adjusted correlations for age, body mass index, and gender. A *p*-value of < 0.05 was considered statistically significant and all data analyses were performed using SAS software (SAS Institute Inc., Cary, NC).

## Results

### Baseline characteristics

The baseline characteristics of eligible participants are presented in Table 1. Out of a total of 2,574 sleep studies reviewed for inclusion in this study, 2092 studies met the eligibility criteria and were analyzed (studies excluded for incomplete NPSG, inadequate/noisy ECG signal, arrhythmias, and participants with CVD at baseline or using beta-blocker or other chronotropic medications). The included studies were analyzed in two different sets. A total of 1,438 files were used as a discovery dataset as a training set to identify the diagnostic threshold before it is validated. The remaining 653 files were analyzed as a validation dataset.

**Table 1:** Participant characteristics from the Sleep Heart Health study (SHHS).

**Characteristic**	**Summary measure**			**Comparison of samples**
	**Total sample** ***n** =* **2,091**	**Discovery sample** ***n** =* **1,438**	**Validation sample** ***n** =* **653**	***p*** **-value**
Age, mean (sd) min-max	60 (10) 39–87	59 (11) 39–87	62 (9) 40–86	< 0.0001
BMI, mean (sd)	28 (5)	28.5 (5)	27.8 (5)	0.0021
Race, *n* (%)				< 0.0001
White	1820 (87)	1278 (89)	542 (83)	
Black	118 (6)	59 (4)	59 (9)	
Other	153 (7)	101 (7)	52 (8)	
Male sex, *n* (%)	888 (42)	631 (44)	257 (39)	0.0525
Smoking, *n* (%)				0.2589
Current	216 (10)	138 (10)	78 (12)	
Former	879 (42)	609 (42)	270 (41)	
Never	991 (48)	688 (48)	303 (47)	
Hypertension, *n* (%)	341 (16)	210 (15)	131 (20)	0.0017
Diabetes, *n* (%)	78 (4)	41 (3)	37 (6)	0.0023
Stroke at baseline, *n* (%)	32 (2)	22 (2)	10 (2)	0.9979
Antihypertensive medications use, excluding chronotropic agents	299 (14)	187 (13)	112 (17)	0.0121
Total recording time, hours	7.8 (1.0)	7.8 (0.9)	7.7 (1.1)	0.4283
Total sleep time, hours	6.2 (1.0)	6.2 (1.0)	6.1 (1.0)	0.1267
AHI, mean (sd) min-max	21 (17) 0.38–116	20 (16) 0.7–116	22 (18) 0.4–102	0.0099
AHI, *n* (%)				0.0351
< 5	219 (10)	148 (10)	71 (11)	
5–15	740 (35)	535 (37)	205 (31)	
> 15	1132 (54)	755 (53)	377 (58)	
SaO2 < 90%, % total sleep time, mean (sd) min-max	2.4 (0–95)	2.4 (0–95)	2.4 (0–80)	0.924
RRDI total, mean (sd) min-max	24 (20) 0–184	24 (19) 0–160	23 (22) 0–184	0.8432
HRAI total, mean (sd) min-max	31 (15) 3.6–118	31 (15) 4.4–102	30 (17) 3.6–118	0.2961
RE RRDI, mean (sd) min-max	8.3 (10) 0–149	8.4 (10) 0–149	8.1 (10) 0–110	0.5162
RE HRAI, mean (sd) min-max	11 (12) 0–127	11 (12) 0.2–127	11 (11) 0–101	0.9267
Arousal index, mean (sd) min-max	21 (11) 3.9–102	21 (10) 3.9–102	21 (11) 4–83	0.7778
Arousal RRDI, mean (sd) min-max	8.7 (6.6) 0–78	8.9 (6.7) 0–78	8.2 (6.4) 0–47	0.0347
Arousal HRAI, mean (sd) min-max	8.9 (6.0) 0.3–62	9.1 (6.0) 0.4–62	8.6 (5.9) 0.3–36	0.1144

AHI, apnea-hypopnea index; HRAI total, the total number of heart rate accelerations divided by the total NPSG recording time in hours; RRDI total, total number of RR interval dips divided by the total NPSG recording time in hours; RE RRDI, respiratory related RRDI; RE HRAI, respiratory related HRAI; SaO_2_, pulse oximetry. Arousal RRDI, arousal related RRDI; Arousal HRAI, arousal related HRAI. Total recording time is calculated as number of hours from light on to light out regardless of sleep or wake stages. Total sleep time is calculated as number of hours from light on to light out that had sleep stages (N1, N2, N3 or REM). SaO_2_ < 90% is defined by the percentile of time that Oxygen saturation is below 90% for the total sleep time.

**Figure 3:**
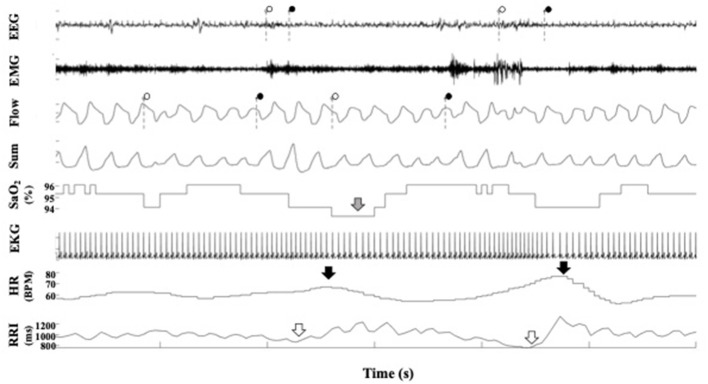
A representative polygraph from a participant in the Sleep Heart Health Study. In the EEG channel, the dotted lines indicate the beginning and ends of arousals scored by SHHS staff. In the flow channel, the dotted lines indicate the beginning and end of respiratory events scored by SHHS staff. In the SaO_2_ channel, the gray arrow indicates a desaturation. In the heart rate channel, the black arrows indicate heart rate accelerations. In the RRI channel, the white arrow indicates RRI dips. EEG, electroencephalogram; EMG, electromyogram; SaO_2_, pulse oximetry; EKG, electrocardiogram; RRI, R-R interval.

Figure 1 is a representative polygraph of one of the participants without SDB (AHI of 3.6 events/h, a total RRDI of 1.7 events/h, and a total HRAI of 4.9 events/h). Figure 2 is a representative analysis of one of the participants who have SDB (AHI of 16.9 events/h a total RRDI of 34.4 events/h and a total HRAI of 45.8 events/h). Figure 3 depicts a polygraph of a 2-min recording segment illustrating HRAs and dips that are associated with respiratory events.

### Agreement with AHI

**Figure 4:**
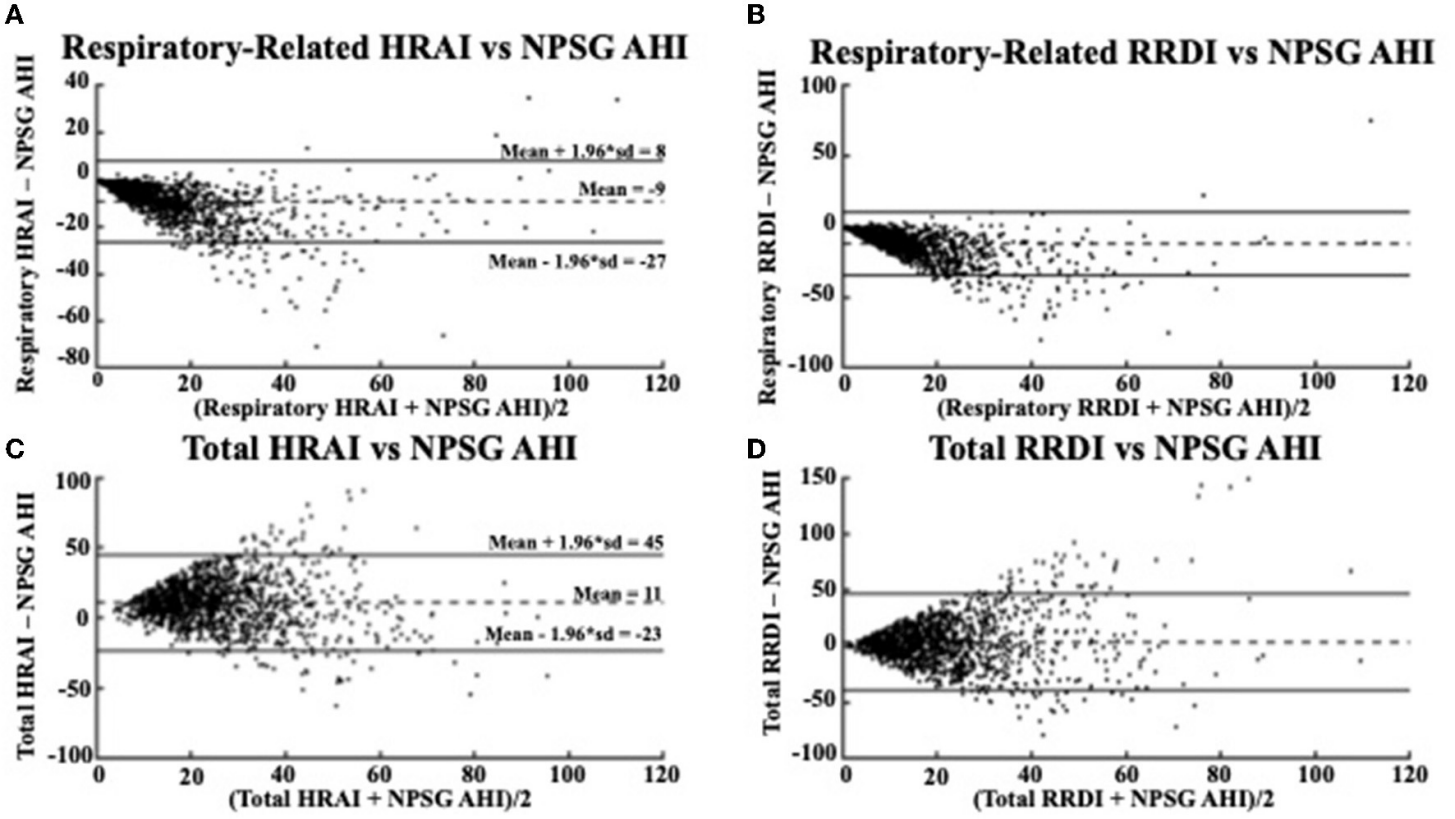
Bland Altman Plots comparing **(A)** respiratory-related HRAI to AHI [ICC = 0.64 (0.61, 0.67)]; **(B)** respiratory-related RRDI to AHI [ICC = 0.38 (0.33, 0.42)]; **(C)** total HRAI to NPSG AHI [ICC = 0.22 (0.17, 0.27)]; and **(D)** total RRDI to NSPG AHI [ICC = 0.22 (0.16, 0.26)] in the discovery dataset. HRAI, heart rate acceleration index; RRDI, RR interval dips index; AHI, apnea–hypopnea index; NPSG, nocturnal polysomnography.

As shown in Table 2, the estimated AHIs using respiratory-related HRAI and respiratory-related RRDI correlated significantly with AHI (*p* < 0.05) and as shown in Figure 4 there is significant agreement between RE HRAI, RE RRDI, and AHI. Further, the Spearman correlation for total HRAI is higher than that of total RRDI after adjusting for age, sex, and body mass index (BMI). These values indicate a strong positive correlation between respiratory-related HRAI and AHI and a moderate correlation between respiratory-related RRDI and AHI (*p* < 0.05).

**Table 2:** Spearman correlations with AHI for total HRAI, total RRDI, RE HRAI, and RE RRDI.

	**Discovery dataset**		**Validation dataset**	
	**Unadjusted**	**Adjusted**	**Unadjusted**	**Adjusted**
Total HRAI	0.30^*^	0.28^*^	0.24^*^	0.26^*^
Total RRDI	0.19^*^	0.18^*^	0.06	0.11
RE HRAI	0.87^*^	0.86^*^	0.83^*^	0.81^*^
RE RRDI	0.71^*^	0.70^*^	0.57	0.56

AHI, apnea-hypopnea index; RRDI, RR interval dips index; HRAI, heart rate acceleration index; RE RRDI, respiratory-related RR interval dips index; RE HRAI, respiratory heart rate acceleration index; PPV, positive predictive value; NPV, negative predictive value. (^*^p < 0.05).

Respiratory-related HRAI showed strong agreement with AHI (intraclass correlation coefficient (ICC: 0.64), whereas respiratory-related RRDI displayed weaker agreement (ICC: 0.38). The Bland-Altman plots in Figure 4 compare the NPSG AHI of the participants with various heart rate-based AHI estimations (respiratory-related HRAI, respiratory-related RRDI, total HRAI, and total RRDI) in the discovery dataset. A low mean negative difference (AHI underestimation) was observed for respiratory-related HRAI and respiratory-related RRDI vs. AHI, although respiratory-related HRAI underestimated it to a lesser degree compared to respiratory-related RRDI (average RE HRAI–NPSG AHI = −9; CI: −27–8 vs. average RE RRDI–NPSG AHI = −12; CI: −35–11). A high mean negative difference (AHI overestimation) was observed for total RRDI and HRAI vs. AHI, although RRDI overestimated it to a lesser degree compared to HRAI (average total RRDI–NPSG AHI = 3; CI: −40–46 vs. average total HRAI–NPSG AHI = 11; CI: −23–45).

**Figure 5:**
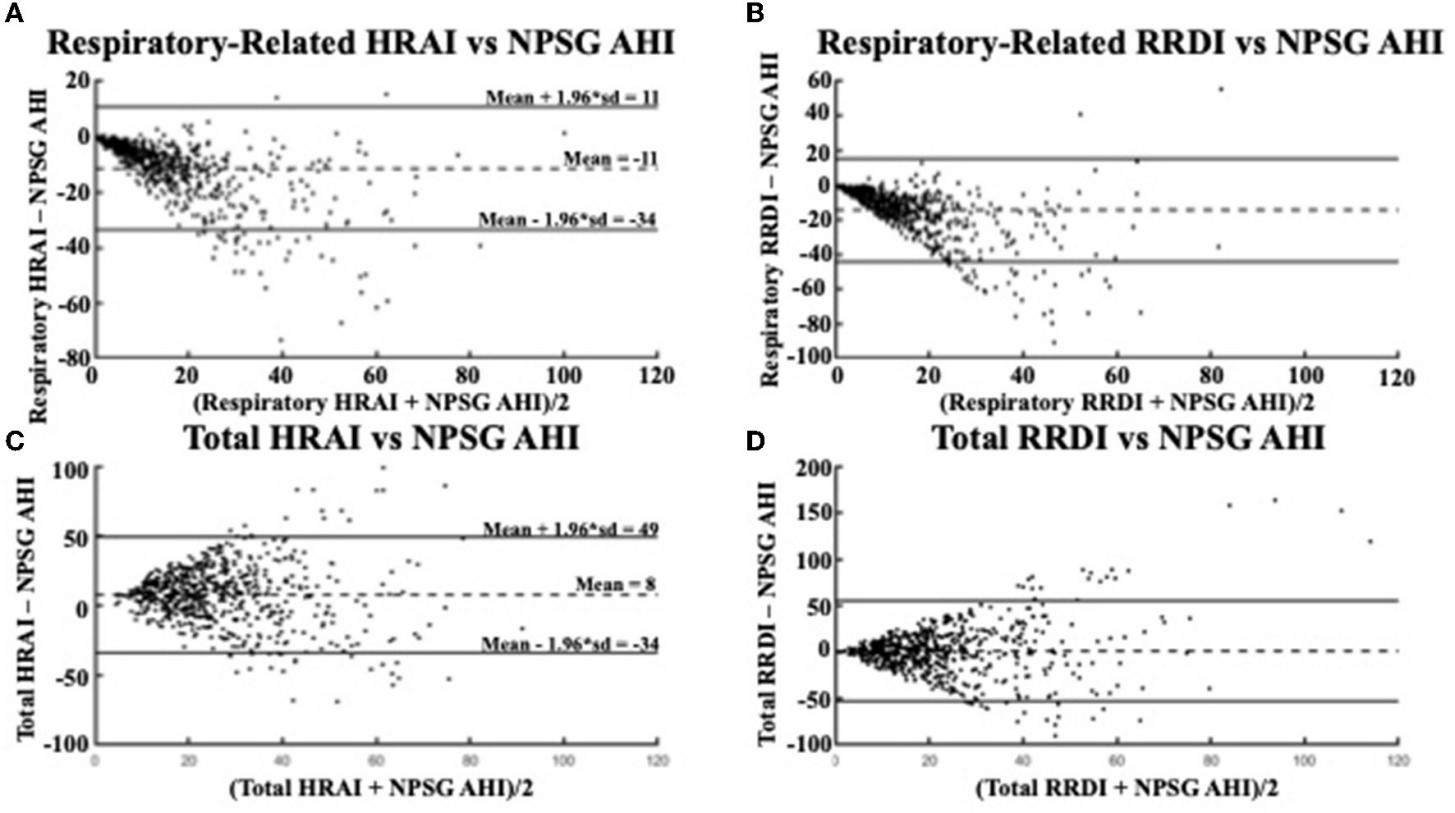
Bland Altman Plots comparing **(A)** respiratory-related HRAI to AHI [ICC =0.51 (0.45, 0.56)]; **(B)** respiratory-related RRDI to AHI [ICC = 0.18 (0.10, 0.25)]; **(C)** total HRAI to NPSG AHI [ICC = 0.19 (0.12, 0.26)]; and **(D)** total RRDI to NSPG AHI [ICC = 0.08 (0.003, 0.16)] in the validation dataset. HRAI, heart rate acceleration index; RRDI, RR interval dips index; AHI, apnea–hypopnea index; NPSG, nocturnal polysomnography.

The Bland-Altman plots in Figure 5 shows a similar relationship in the validation dataset for the respiratory-related measures and AHI (average RE HRAI–NPSG = −11; CI: −34–11 vs. average RE RRDI–NPSG = −14; CI: −44–15). The relationship between total HRAI/RRDI and AHI was also similar in the (average total HRAI–NPSG AHI = 8; CI: −35–49 vs. average total RRDI–NPSG AHI = 1; CI: −53–55). The ICC values were small for both HRAI and RRDI in the discovery dataset (ICC = 0.22 for HRAI vs. AHI and ICC = 0.22 for HRAI vs. AHI) and were even smaller in the validation dataset (ICC = 0.19 for HRAI vs. AHI and ICC = 0.08 for HRAI vs. AHI).

### Diagnostic performance

When the respiratory-related HRAI (≥5 events/h) is used there is a strong diagnostic ability (78, 87, 81, and 56% agreement for traditional AHI cutoffs 5, 10, 15, and 30 events/h, respectively) as depicted in Table 3. In addition, a high level of sensitivity was found with respiratory-related HRAI (≥5 events/h) with traditional AHI cutoffs, 5, 10, 15, and 30 events/h, respectively (75, 87, 92, 97%, respectively). RE RRDI (≥5 events/h) showed less modest agreement (59, 70, 73, 63%) for traditional AHI cutoffs 5, 10, 15, and 30 events/h, respectively, and higher specificity compared to HRAI.

**Table 3:** Diagnostic testing for RE RRDI and HRAI as a metric of estimated AHI ≥ 5 for the validation dataset.

	**AHI cut off (RE RRDI** ≥**5)**				**AHI cut off (RE HRAI** ≥**5)**			
	**AHI 5**	**AHI 10**	**AHI 15**	**AHI 30**	**AHI 5**	**AHI 10**	**AHI 15**	**AHI 30**
Sensitivity, %	54%	63%	69%	71%	75%	87%	92%	97%
Specificity, %	100%	88%	79%	59%	100%	85%	67%	43%
PPV, %	100%	93%	82%	36%	100%	94%	79%	36%
NPV, %	21%	48%	65%	86%	33%	72%	86%	98%
Agreement, %	59%	70%	73%	63%	78%	87%	81%	56%
Kappa	0.21	0.40	0.46	0.22	0.40	0.68	0.61	0.25

AHI, apnea-hypopnea index; RE RRDI, respiratory RR interval dips index; RE HRAI, respiratory heart rate acceleration index; PPV, positive predictive value; NPV, negative predictive value.

Table 4 shows the four-class confusion matrix comparing the classification agreement AHI with the classification of the RE HRAI at different thresholds in the validation dataset. Furthermore, dividing the validation dataset into two subgroups based on age < 55 and ≥55 years respectively showed a similar and strong diagnostic ability for RE HRAI (79, 87, 83, and 56% agreement for traditional AHI cutoffs 5, 10, 15, and 30 events/h, respectively) in the age group of ≥55 years of age and (76, 87, 78, and 58% agreement for traditional AHI cutoffs 5, 10, and 15, and 30 events/h, respectively) in the subgroup < 55 years of age as depicted in Supplementary Tables S1, S2.

**Table 4:** Four–class confusion matrix showing classification agreement of AHI and respiratory-related HRAI in the validation dataset.

	**RE HRAI severity**				
**AHI**	**RE HRAI**<**5**	**5** ≤ **RE HRAI**<**10**	**10** ≤ **RE HRAI**<**15**	**15** ≤ **RE HRAI**<**30**	**30** ≤ **RE HRAI**
AHI < 5	71	0	0	0	0
5 ≤ AHI < 10	84	27	1	0	0
10 ≤ AHI < 15	30	54	8	1	0
15 ≤ AHI	25	87	65	39	0
30 ≤ AHI	5	23	29	57	47

AHI, apnea-hypopnea index; RE HRAI, respiratory-related heart rate acceleration index.

**Figure 6:**
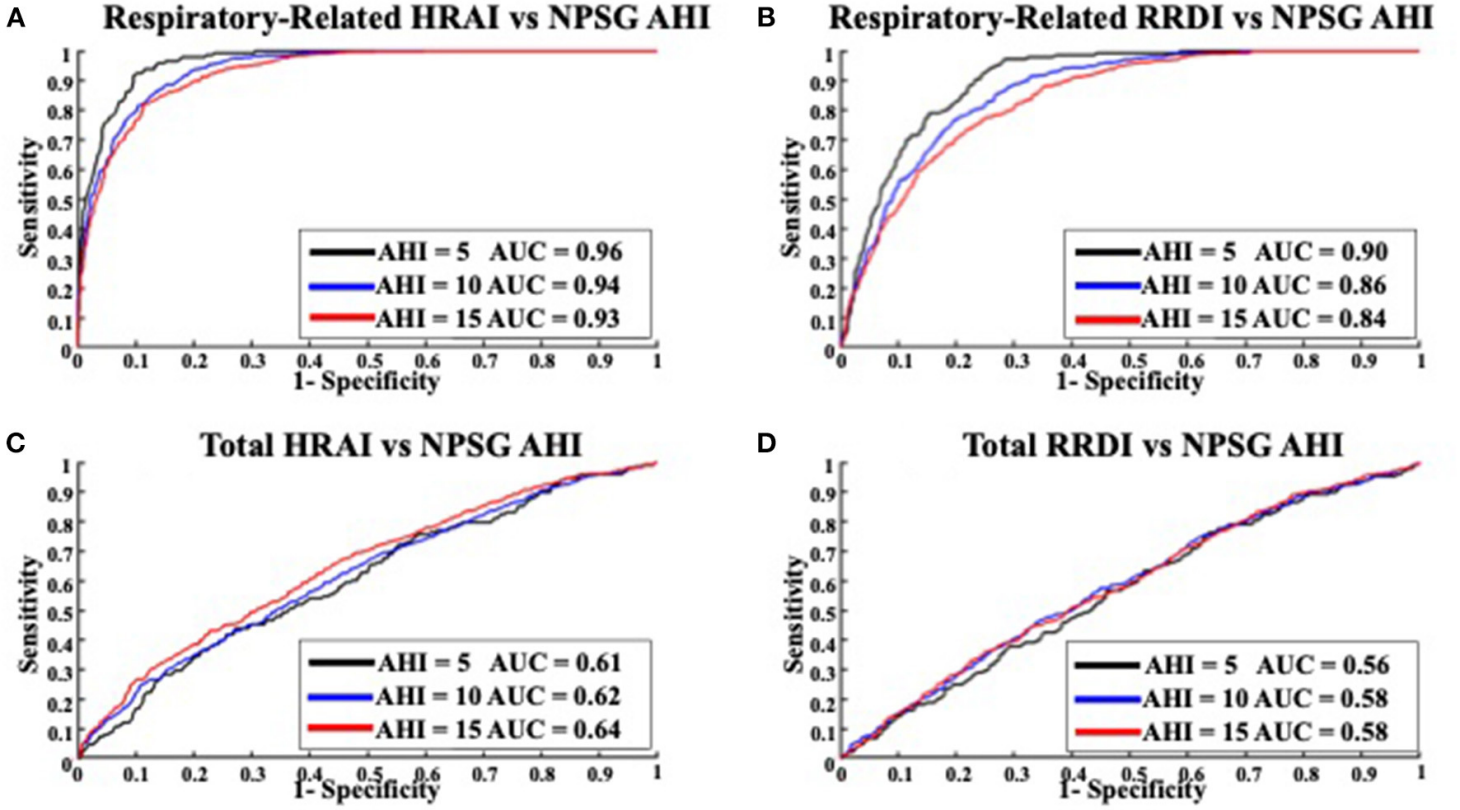
Receiver operating characteristic curves for **(A)** respiratory-related HRAI to AHI; **(B)** respiratory-related RRDI to AHI; **(C)** total HRAI to NPSG AHI; and **(D)** total RRDI to NSPG AHI in the discovery dataset. HRAI, heart rate acceleration index; RRDI, RR interval dips index; AHI, apnea-hypopnea index; NPSG, nocturnal polysomnography.

**Figure 7:**
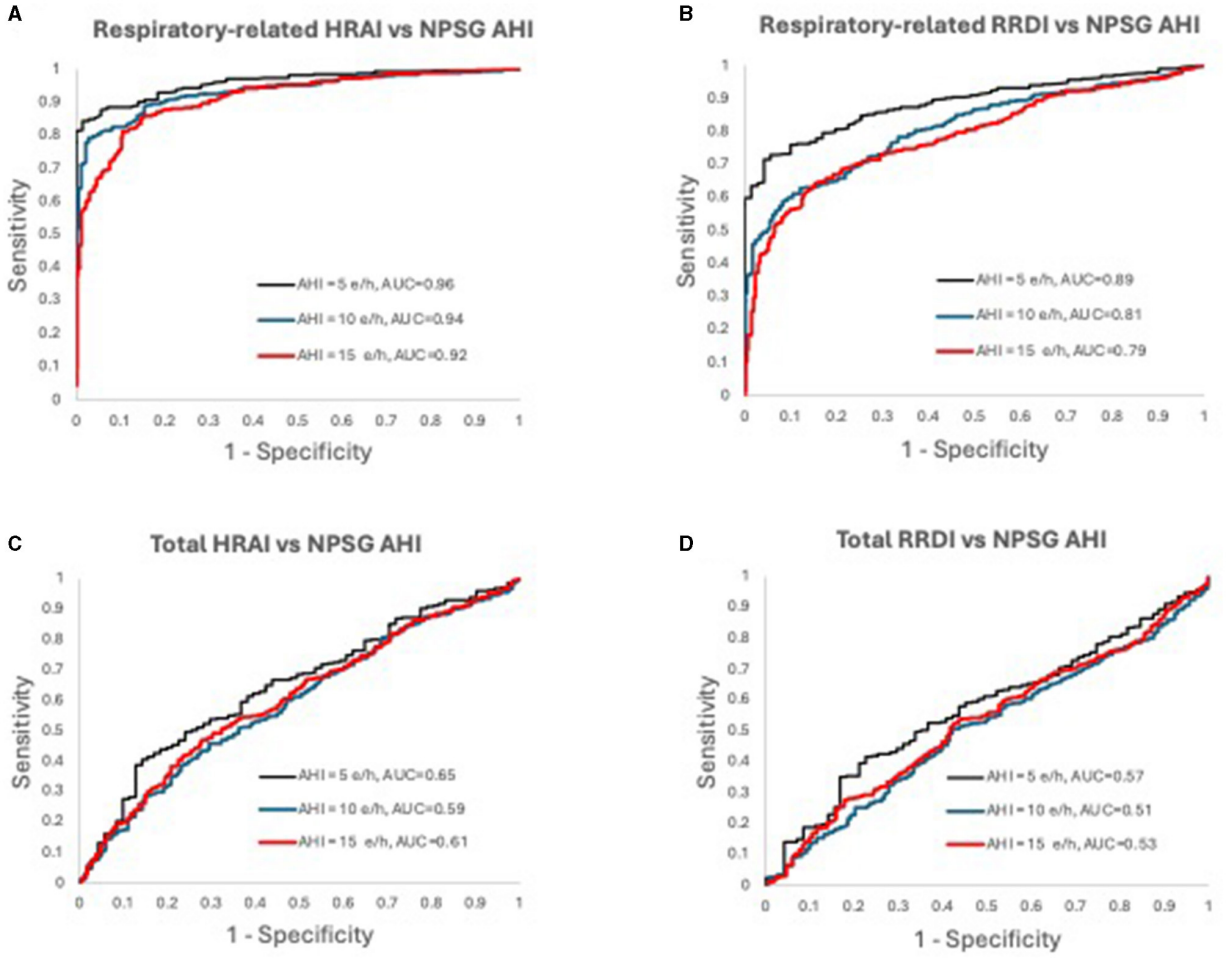
Receiver operating characteristic curves for **(A)** respiratory-related HRAI to AHI; **(B)** respiratory-related RRDI to AHI; **(C)** total HRAI to NPSG AHI; and **(D)** total RRDI to NSPG AHI in the validation dataset. HRAI, heart rate acceleration index; RRDI, RR interval dips index; AHI, apnea-hypopnea index; NPSG, nocturnal polysomnography.

Figures 6, 7 display the receiver operating characteristic curves for respiratory-related HRAI/RRDI and total HRAI/RRDI with three AHI cutoffs for the diagnosis of SDB in the discovery and validation datasets, respectively. The corresponding AUC for each respiratory-related attribute diagnostic cutoff was high in both datasets, with decreasing scores as the AHI cutoff increased (see Supplementary Figure S6). However, the AUC scores were significantly lower for total HRAI and RRDI. Using RE RRDI and RE HRAI values to predict AHI ≥5 events/h revealed a sensitivity of 0.99 for both indices and the agreement was 63 and 78% for RE RRDI and RE HRAI respectively. At the cutoff of AHI ≥5 events/h only, the ROC revealed AUCs of 0.90 and 0.96 for RE RRDI and RE HRAI, respectively.

## Discussion

### Summary of findings

This study provides several important findings: (1) heart rate measurements derived from ECG and pulse oximeter signals are strongly correlated with AHI (derived from gold standard NPSG); (2) Nocturnal pulse rate changes provide a greater diagnostic ability than ECG-derived metrics with higher sensitivity and agreement; (3) Nocturnal respiratory related pulse-rate analytic approaches are capable of reliably identifying adults with SDB, using AHI clinical cutoff value (AHI ≥5 events/hour), while also allowing for accurate estimates of NPSG derived AHI and thus could lead the way to find an effective, non-invasive preliminary screening tool to detect the presence of SDB.

The gold standard for diagnosing SDB currently is an attended in-laboratory polysomnography, involving the simultaneous recording of physiological measures, including EEG, EOG, chin and leg EMG, ECG, nasal–oral thermistors, and pulse oximetry (Ucak et al., 2021). In contrast, home sleep apnea testing (HSAT) is less intensive, unattended, and may be more representative of an individual's typical sleep pattern. Both types of tests require manual scoring and may be affected by subjective scorer bias (Collop, 2002). Therefore, there is a critical need to develop automated, valid, and reliable developing techniques using more readily available equipment and automated analysis techniques should therefore be prioritized to circumvent these limitations as well as to allow for more accessible and affordable SDB screening.

Previous research has investigated the use of heart rate measurements from single-lead ECG to detect OSA. De Chazal et al. (2003) extracted heart rate variability (HRV) time and frequency domain features and were able to differentiate apneic recording segments from normal segments with a success rate between 90.5 to 100%. A more recent investigation performed by Zarei and Asl (2020) utilized more advanced methods that incorporated multiple HRV parameters and achieved 93.3% accuracy in detecting respiratory events. In a similar study, Rahman et al. (2018) used a supervised classification technique to categorize patients as having either severe or non-severe sleep apnea with a high degree of success (accuracy 87.5%, sensitivity 100%, and specificity 83.3%). The findings of our study were comparable to these previous investigations but employed time-domain features instead of frequency-domain features. Additionally, our sample size was considerably larger than that of all three of these studies as we analyzed over 2,000 NPSGs whereas none of these analyzed more than 200 NPSGs. The percent agreement for respiratory-related HRAI ranged from 67 to 87%, whereas that for respiratory-related RRDI ranged from 54 to 63%. In our study, at all cutoff values assessed for respiratory-related HRAI, the sensitivity was exceptional (79 to 100% for AHI cutoff 15 to 5 events, respectively as shown in Table 3), indicating that our algorithm can be highly effective in ruling out individuals without SDB. With the higher cutoff values, the sensitivity was reduced, meaning that these cutoffs would be less effective in identifying individuals with SDB.

There are also some similarities between our approach and that of cardiopulmonary coupling (CPC). CPC also uses RRI data, but it employs frequency domain analysis instead of time domain analysis to convert periods of RRI into various frequency coupling bands (Thomas et al., 2018). These frequency coupling bands include high-frequency coupling (HFC) which corresponds to periods of stable sleep, low-frequency coupling (LFC) which corresponds to periods of fragmented sleep, elevated low-frequency coupling (e-LFC) which is correlated to periods of the obstructive airway, and sustained chemoreflex effects on respiration and very low-frequency coupling (VLFC) which corresponds to periods of wake or REM sleep. It has been demonstrated that HFC showed higher intraclass coefficients in healthy participants than in those with sleep apnea (Thomas et al., 2018). Ma et al. (2020) used CPC to derive a respiratory event index (CPC-REI) and found that this measure was positively correlated with AHI (r = 0.851, *p* < 0.001). This method differs from our method in the way that the respiratory events are defined, as those in CPC do not necessarily align with respiratory events scored in the same way as the respiratory-related RRI dips and respiratory-related heart rate accelerations.

### Automated signal analysis

Our work contributes to the exciting and ever-advancing area of automated analysis in sleep research. Other groups have used machine learning algorithms to estimate SDB-related metrics from a subset of NPSG signals. For example, Bozkurt et al. (2019) developed a machine-learning-based apnea detection algorithm using data from Photoplethysmography (PPG) signal and HRV. After using digital filters to clean PPG signals, a real-time embedded four-feature PPG system was able to operate with an accuracy of 93.8% and thus was an effective method for diagnosing respiratory events. Another investigation by Hornero et al. (2017), used a convolutional neural network to predict AHI from only the oxygen saturation signal in pediatric patients. Their model was very accurate and increased as the AHI cutoff increased from 1 events/h to 10 events/h. This differs from our two models which were more accurate at lower AHI cutoffs, meaning that our system may be more effective in diagnosing those with mild SDB. Their model also differs from ours by not relying on changes in the saturation signal to align with respiratory events. Instead, their model was trained on 589 PSGs with scored AHI and applied to 3,602 other PSGs. Urtnasan et al. (2018), similarly used a convolutional neural network to classify 10-second epochs as either having obstructive apnea or not having obstructive apnea by analyzing the raw ECG signal alone. They were able to achieve 96% sensitivity and specificity in their test set after forming the model in a training set of 63 patients. A limitation of this model was that it was only used to identify those with obstructive apneas.

Recent studies also explored the role of single-lead ECG in detecting cortical arousals from home sleep studies using deep learning techniques and found an AUC score of 0.93 (Li et al., 2020). Likewise, Lachapelle et al. (2019) used heart rate changes to predict cortical arousals. As previously mentioned, the inability to detect cortical arousals is a weakness of home sleep apnea tests. Lachapelle et al. found that they could improve agreement between home sleep apnea tests AHI and full NPSG AHI if they included non-scored hypopneas that were accompanied by increases in heart rate (mean difference in AHI 11.2 events/hr vs. 7.1 events/hr, *p* = 0.01). The objective of their study differed from ours, but their results demonstrate the potential benefits of studying heart rate changes associated with respiratory events. Nevertheless, in our study, we found that approximately a third of the total heart rate changes (RRDI and HRAI) were related to cortical arousals (as outlined in Table 1 and Figure 2), indicating the importance of arousals in the overall estimated score for severity of SDB. In addition, it suggests that this technique may enhance the diagnostic yield of sleep studies (such as HSAT) that do not score arousals to provide more accurate respiratory events scores.

There have been many studies that have used the oxygen desaturation index (ODI) as a predictor for AHI. Golpe et al. compared 4% ODI to AHI and found that a cutoff value of ODI = 31.4 was most accurate for predicting an AHI value of 15 events/hour or more (Lachapelle et al., 2019). This method exhibited a sensitivity of 32% and a specificity of 97%, indicating that it could be used to exclude severe disease but not necessarily rule it in. Hang et al. used a more sophisticated algorithm that employed a support vector machine to create a model that used many different features from the oxygen saturation signal derived from a pulse oximeter to predict AHI (Lachapelle et al., 2019). They were able to achieve a sensitivity of 86% and a specificity of 93% when diagnosing patients with severe sleep apnea (AHI = 30 events/h). The methods of predicting AHI from ODI have value, though our model has the advantage of having a very high specificity at the mild cutoff point, allowing us to minimize false negatives for the detection of the presence of sleep apnea.

### Clinical implications

The findings of this study suggest a clear association between heart rate changes detected through ECG and pulse oximeter signals and respiratory events. The strong correlations between respiratory-related heart rate changes and AHI suggest that automated analysis using signals from sleep studies can accurately identify respiratory events using signals such as pulse rate (derived from pulse oximetry signal). These observations are a major step in the direction of developing a simple heart rate-based algorithm to diagnose SDB. Such a breakthrough would have the potential to greatly decrease screening costs and allow for a much greater pool of individuals to be assessed for SDB.

### Limitations

There are several limitations to this work, the most notable of which was poor ECG signal quality in some studies. Studies with more than 10% of the signal that could not be analyzed due to poor signal quality were excluded. This is a potential weakness of using respiratory-related RRDI as a surrogate for AHI, but it also demonstrates a potential strength of using HRAI. Another limitation was that this method of analysis is not suitable for patients who have arrhythmias or are on certain medications that influence heart rate (Miyatani et al., 2018). Patients who have atrial fibrillation or multiple premature ventricular contractions, for example, have heart rates that are more variable and will have artificially higher RRDIs and HRAIs. Despite those differences, we observed that both RRDIs and HRAIs correlate strongly with each other (with a correlation coefficient = 0.83; *p* < 0.0001). However, based on this study HRAI performs better diagnostically. Our explanation is that the signal is smoother and gets affected less by the presence of ectopic beats or taking medications or chronotropic agents such as beta-blockers (ongoing study). In addition, these two signals are not the same in term of sampling rate, thresholds, or circulation time which explain the delay noticed in Figure 2. It should also be noted that the participants in the SHHS database were overwhelmingly Caucasian, and future studies involving this type of analysis in additional cohorts are warranted. There are also some inherent weaknesses in our findings due to the data being obtained from a retrospective cohort study. The original SHHS study was not designed specifically for this type of analysis. Cohort studies are prone to bias, but this was accounted for by implementing the automated analysis of the sleep studies which were reviewed visually to ensure that signal quality was acceptable and that arrhythmias were not present.

Another major limitation of this work is that the respiratory-related HRAI and RRDI indices, which have greater predictive ability than their total HRAI/RRDI counterparts, require events to be manually scored. The goal of this work is to make the diagnosis of sleep apnea simpler and less expensive. Recent studies assessed the ability to detect ECG-derived respiration (EDR) and showed promising results and strong agreement with cardiopulmonary coupling such as that was used in this study (De Chazal et al., 2003; Zheng et al., 2016). Hence, it may allow combing these techniques in the future to assist in automatically detecting these events from simplified recording. Further studies in this area could greatly assist in the development of these algorithms.

## Conclusion

In conclusion, our data showed that nocturnal heart rate changes (HRAI) derived from ECG and pulse rate correlate strongly with SDB severity. However, pulse rate changes provide a greater diagnostic ability than ECG-derived metrics with higher accuracy that can provide accurate estimates of AHI, especially in cases of limited settings such as using home sleep apnea testing. Future studies are needed to validate this method in other independent samples and representative clinical settings. Work in this area should be continued to enhance automated heart rate analysis to allow for accurate prediction of AHI using only simple equipment.

## Data availability statement

The original contributions presented in the study are included in the article/Supplementary material, further inquiries can be directed to the corresponding author.

## Ethics statement

The protocol was approved by WSU for secondary analysis of SHHS de-identified dataset accessed from the National Sleep Research Resource (NSRR).

## Author contributions

All authors listed have made a substantial, direct, and intellectual contribution to the work and approved it for publication.
