# Corrigendum: Role of automated detection of respiratory related heart rate changes in the diagnosis of sleep disordered breathing

**DOI:** 10.3389/frsle.2024.1452220

**Published:** 2024-07-29

**Authors:** Scott Maresh, Adhithi Keerthana Athikumar, Nabila Ahmed, Shivapriya Chandu, Joel L. Prowting, Layth Tumah, Abed A. Najjar, Hamza Khan, Muna Sankari, Oluwatobi Lasisi, Laurel A. Ravelo, Paul E. Peppard, M. Safwan Badr, Abdulghani Sankari

**Affiliations:** ^1^Sleep Research Laboratory, John D. Dingell Veterans Affairs Medical Center, Detroit, MI, United States; ^2^Department of Internal Medicine, Wayne State University-School of Medicine, Detroit, MI, United States; ^3^Department of Kinesiology and Health Science, York University, Toronto, ON, Canada; ^4^Population Health Sciences, University of Wisconsin-Madison, Madison, WI, United States; ^5^Department of Medical Education, Ascension Providence Hospital, Southfield, MI, United States

**Keywords:** sleep, heart rate, R-R interval (RRI), pulse oximeter, ECG, polysomnography, sleep apnea and cardiovascular disease

In the published article, there was an error in the legend for **Figure 4** as published. The corrected legend appears below.

**Figure 4**. Bland Altman Plots comparing **(A)** respiratory-related HRAI to AHI [ICC = 0.64 (0.61, 0.67)]; **(B)** respiratory-related RRDI to AHI [ICC = 0.38 (0.33, 0.42)]; **(C)** total HRAI to NPSG AHI [ICC = 0.22 (0.17, 0.27)]; and **(D)** total RRDI to NSPG AHI [ICC = 0.22 (0.16, 0.26)] in the discovery dataset. HRAI, heart rate acceleration index; RRDI, RR interval dips index; AHI, apnea–hypopnea index; NPSG, nocturnal polysomnography.

In the published article, there was an error in the legend for **Figure 5** as published. The corrected legend appears below.

**Figure 5**. Bland Altman Plots comparing **(A)** respiratory-related HRAI to AHI [ICC =0.51 (0.45, 0.56)]; **(B)** respiratory-related RRDI to AHI [ICC = 0.18 (0.10, 0.25)]; **(C)** total HRAI to NPSG AHI [ICC = 0.19 (0.12, 0.26)]; and **(D)** total RRDI to NSPG AHI [ICC = 0.08 (0.003, 0.16)] in the validation dataset. HRAI, heart rate acceleration index; RRDI, RR interval dips index; AHI, apnea–hypopnea index; NPSG, nocturnal polysomnography.

In the published article, there was an error in the legend for **Figure 6** as published. The corrected legend appears below.

**Figure 6**. Receiver operating characteristic curves for **(A)** respiratory-related HRAI to AHI; **(B)** respiratory-related RRDI to AHI; **(C)** total HRAI to NPSG AHI; and **(D)** total RRDI to NSPG AHI in the discovery dataset. HRAI, heart rate acceleration index; RRDI, RR interval dips index; AHI, apnea-hypopnea index; NPSG, nocturnal polysomnography”.

In Figure 7 the graphs were incorrectly ROC curves plotted. Below are the corrected ROC curves.

**Figure 7:**
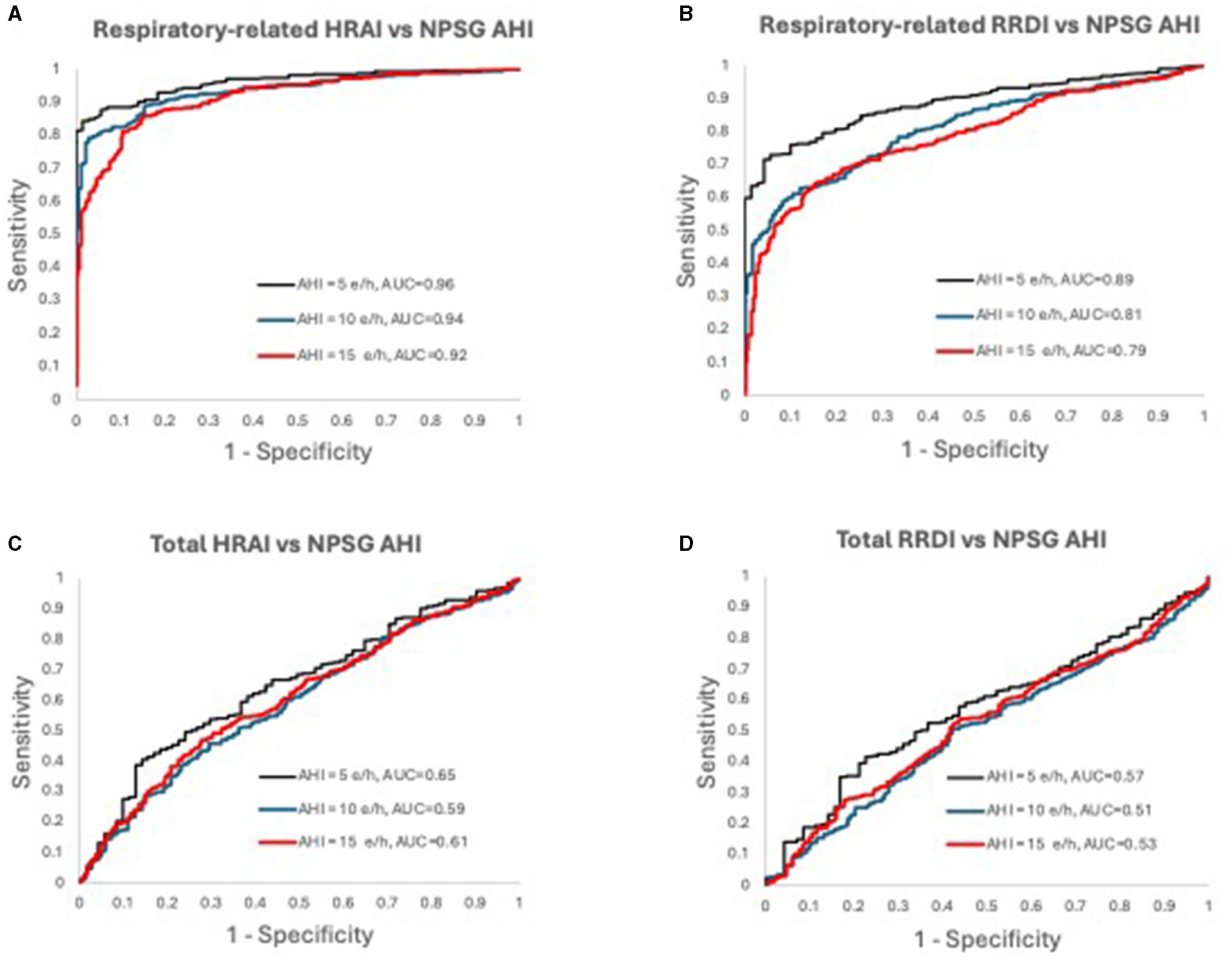
Receiver operating characteristic curves for **(A)** respiratory-related HRAI to AHI; **(B)** respiratory-related RRDI to AHI; **(C)** total HRAI to NPSG AHI; and **(D)** total RRDI to NSPG AHI in the validation dataset. HRAI, heart rate acceleration index; RRDI, RR interval dips index; AHI, apnea-hypopnea index; NPSG, nocturnal polysomnography.

The estimated sensitivity, specificity, PPV, and NPV were listed incorrectly. The corrected Table 3 and its caption appear below.

**Table 3:** Diagnostic testing for RE RRDI and HRAI as a metric of estimated AHI ≥ 5 for the validation dataset.

	**AHI cut off (RE RRDI** ≥**5)**				**AHI cut off (RE HRAI** ≥**5)**			
	**AHI 5**	**AHI 10**	**AHI 15**	**AHI 30**	**AHI 5**	**AHI 10**	**AHI 15**	**AHI 30**
Sensitivity, %	54%	63%	69%	71%	75%	87%	92%	97%
Specificity, %	100%	88%	79%	59%	100%	85%	67%	43%
PPV, %	100%	93%	82%	36%	100%	94%	79%	36%
NPV, %	21%	48%	65%	86%	33%	72%	86%	98%
Agreement, %	59%	70%	73%	63%	78%	87%	81%	56%
Kappa	0.21	0.40	0.46	0.22	0.40	0.68	0.61	0.25

AHI, apnea-hypopnea index; RE RRDI, respiratory RR interval dips index; RE HRAI, respiratory heart rate acceleration index; PPV, positive predictive value; NPV, negative predictive value.

In the published article, there was an error in the Supplementary section for some sensitivity and specificity values. The Supplementary Tables S1, S2 has been updated in the original article.

In the published article, there was an error. **Figure 4** and **Table 2** are erroneously stated as correlating with AHI.

A correction has been made to **Results**, *Agreement with AHI*, paragraph one. This sentence previously stated:

“As shown in **Table 2** and **Figure 4**, the estimated AHIs using respiratory-related HRAI and respiratory-related RRDI correlated significantly with AHI (*p* < 0.05).”

The corrected sentence appears below:

“As shown in **Table 2**, the estimated AHIs using respiratory-related HRAI and respiratory-related RRDI correlated significantly with AHI (*p* < 0.05) and as shown in **Figure 4** there is significant agreement between RE HRAI, RE RRDI, and AHI.”

In the published article, there was an error. A citation for **Figure 5** should be changed to **Figure 4**.

A correction has been made to **Results**, *Agreement with AHI*, paragraph two. This sentence previously stated:

“The Bland-Altman plots in **Figure 5** compare the NPSG AHI of the participants with various heart rate-based AHI estimations (respiratory-related HRAI, respiratory-related RRDI, total HRAI, and total RRDI) in the discovery dataset.”

The corrected sentence appears below:

“The Bland-Altman plots in **Figure 4** compare the NPSG AHI of the participants with various heart rate-based AHI estimations (respiratory-related HRAI, respiratory-related RRDI, total HRAI, and total RRDI) in the discovery dataset.”

In the published article, there was an error. A citation for **Figure 6** should be changed to **Figure 5**.

A correction has been made to **Results**, *Agreement with AHI*, paragraph three. This sentence previously stated:

“The Bland-Altman plots in **Figure 6** shows a similar relationship in the validation dataset for the respiratory-related measures and AHI (average RE HRAI–NPSG = −11; CI: −34–11 vs. average RE RRDI–NPSG = −14; CI: −44–15).”

The corrected sentence appears below:

“The Bland-Altman plots in **Figure 5** shows a similar relationship in the validation dataset for the respiratory-related measures and AHI (average RE HRAI–NPSG = −11; CI: −34–11 vs. average RE RRDI–NPSG = −14; CI: −44–15).”

In the published article, there was an error in the levels of sensitivity and specificity.

A correction has been made to **Results**, *Diagnostic performance*, paragraph one. These sentences previously stated:

“In addition, a high level of sensitivity was found with respiratory-related HRAI (≥5 events/h) with traditional AHI cutoffs 5, 10, 15, and 30 events/h, respectively (100, 94, 79, and 36%, respectively). RE RRDI (≥5 events/h) showed less modest agreement (59, 70, 73, and 63%) for traditional AHI cutoffs 5, 10, 15, and 30 events/h, respectively, and lower specificity compared to HRAI, especially at a high AHI cutoff of 15 events/hour.”

The corrected sentences appear below:

“In addition, a high level of sensitivity was found with respiratory-related HRAI (≥5 events/h) with traditional AHI cutoffs, 5, 10, 15, and 30 events/h, respectively (75, 87, 92, 97%, respectively). RE RRDI (≥5 events/h) showed less modest agreement (59, 70, 73, 63%) for traditional AHI cutoffs 5, 10, 15, and 30 events/h, respectively, and higher specificity compared to HRAI.”

In the published article, there was an error. A citation for **Figure 6** was excluded.

A correction has been made to **Results**, *Diagnostic performance*, third paragraph. This sentence previously stated:

“Figure 7 displays the receiver operating characteristic curves for respiratory-related HRAI/RRDI and total HRAI/RRDI with three AHI cutoffs for the diagnosis of SDB in validation datasets, respectively.”

The corrected sentence appears below:

“**Figures 6**, 7 display the receiver operating characteristic curves for respiratory-related HRAI/RRDI and total HRAI/RRDI with three AHI cutoffs for the diagnosis of SDB in the discovery and validation datasets, respectively.”

The authors apologize for these errors and state that this does not change the scientific conclusions of the article in any way. The original article has been updated.

