# Supplementary material for: Role of automated detection of respiratory related heart rate changes in the diagnosis of sleep disordered breathing
Source: Front Sleep. 2023 Jun 22;2:1162652. doi: 10.3389/frsle.2023.1162652 (PMC12713967; doi:10.3389/frsle.2023.1162652)
Supplement: Supplementary file 1 [file Data_Sheet_1.docx]

###### Supplements File

###### Role of Automated Detection of Respiratory Related Heart Rate Changes in the Diagnosis of Sleep Disordered Breathing.

###### Scott Maresh, M.S. (1,2)

Adhithi Keerthana Athikumar (2)

Nabila Ahmed (2)

Shivapriya Chandu (2)

Joel L. Prowting, M.S., CSCS (3)

Layth Tumah, M.D. (2)

Abed A. Najjar (2)

Hamza Khan, B.A. (2)

Muna Sankari (2)

Oluwatobi Lasisi, M.D. (1,2)

Laurel Ravelo, M.S. (4)

Paul E. Peppard, Ph.D. (4)

M Safwan Badr, M.D., M.B.A. (1,2)

*Abdulghani Sankari, M.D., Ph.D. (1,2,5)

###### (1) Sleep Research Laboratory, John D. Dingell Veterans Affairs Medical Center, Detroit, MI

###### (2) Department of Internal Medicine, Wayne State University-School of Medicine, Detroit, MI

(3) Department of Kinesiology and Health Science, York University, Toronto, ON

(4) Population Health Sciences, University of Wisconsin-Madison, Madison, WI

(5) Department of Medical Education, Ascension Providence Hospital, Southfield, MI

###### * Address correspondence to: Abdulghani Sankari, M.D., Ph.D., Division of Pulmonary, Critical Care and Sleep Medicine, 3990 John R, 3-Hudson, Detroit, MI 48201; Tel: (313) 745-6033; Fax: (313) 745-8725; Email: asankari@wayne.edu

FIG. 1S illustrates an example system 100 for analyzing whether a patient has SDB. The system includes an electrocardiography (ECG) device 102 configured to create RRI data 104, and a pulse oximeter 106 configured to create oxygen saturation (SaO2) data 108. The system 100 further includes a computing device 110 having a processor 112, a memory 114, a storage 116, and an input/output interface 120. The analysis application 118 may be an application included on the storage 116 of the computing device 110. The computing device 110 receives the RRI data 104 and the oxygen saturation data 108 (e.g., via a transceiver 112), and executes the analysis application 118 to create results 122. The results 122 may be provided to a display 124 for review.

The analysis application 118 includes instructions that, when loaded into the memory 114 and executed by the processor 112, cause the computing device 110 to utilize the RRI data 104 and the SaO2 data to determine results 122 indicative of whether or not a patient has SDB. Specific examples of these instructions are described in detail with reference to the processes below.

FIG. 2S illustrates an example process 200 for determining whether a patient has SDB according to the analysis of SaO2 data 108 and analysis of RRI data 104. As discussed in detail, the analysis application 118 detects sleep-disordered breathing using ECG tracing and beat-to-beat heart rate variability. Respiratory events are typically followed by a drop in RRI and a desaturation. Accordingly, the analysis application 118 analyzes changes in R-R interval (RRI) length (heart rate) and desaturations during sleep.

At 202, the analysis application 118 receives the SaO2 data 108 and RRI data 104. For example, the data may be received from the ECG 102 and Pulse Oximeter 106 from a connected patient. In other examples, the SaO2 data 108 and RRI data 104 may be received from storage, e.g., having been collected from a previous recording of patient ECG tracing and beat-to-beat heart rate variability. In some examples, the analysis application 118 may perform preprocessing of the RRI data 104. This preprocessing may be done to clean the data for further processing. As some examples, the preprocessing may include normalizing the heights of the raw RRI values, removing short artifacts, and adding missing beats, if necessary. In other examples, the analysis application 118 may receive RRI data 104 in which the raw RRI values have been manually reviewed for consistency. In some examples, one aspect of cleaning the RRI data 104 includes removing indexed RRI data points from the RRI data 104. This removal may be utilized later in determining the total amount of RRI data 104 that was processed.

At 204, the analysis application 118 analyzes the SaO2 data 108 to identify desaturation events. Further aspects of the analysis are illustrated with reference to process 300 of FIG. 3. Referring to FIG. 3S, at 302 the analysis application 118 removes low SaO2 data 108 values. (e.g., those that are less than 50%). These values may be removed because these are likely to be artifacts, as values this low are rare or indicative of other health-related issues. In other examples, the threshold percentage for removal could be even higher, e.g., 60%, 70%, or even 80%. At 304, the analysis application 118 divides the SaO2 data 108 values into intervals (e.g., one-second intervals). At 306, the analysis application 118 determines an average value for each of the intervals. At 308, the analysis application 118 deletes data points from the SaO2 data 108 that are more than a predefined amount below the average (e.g., that are 0.1% smaller than the average in an example). These removals may also be done to remove data artifacts, although this removal may not necessarily be directly tied to a specific physiological effect.

The analysis application 118 then cycles through the intervals. At 310, the analysis application 118 begins at the first interval. At 312, the analysis application 118 determines the maximum value in the next set of intervals (e.g., the minimum value across the next 60 intervals in an example). For instance, the analysis application 118 may review the next sample's data until the time of the reviewed data is at least 60 intervals in length greater than the timestamp of the current maximum value (or the maximum amount available if fewer data than 60 intervals remain) to find a minimum. It should be noted that the use of a timeframe of 60 intervals is only one example, and other values, such as 30 or 90 times the interval length may be used. The analysis application 118 also calculates the desaturation for the interval at 316. For example, analysis application 118 may subtract the maximum value of the interval from the minimum value identified for the set of intervals. If at 316 the analysis application 118 determines that the desaturation is at least a predefined value (e.g., 1%), the analysis application 118 proceeds to 320 to record or mark the SaO2 levels and time information for later processing. After 318 or 320, the analysis application 118 proceeds to 322 to determine whether additional intervals remain. If so, the analysis application 118 transitions to 324 to increment to the next interval, and to operation 312 to continue cycling through the intervals. If no further intervals remain, process 300 ends, and the flow return to process 200.

Referring back to FIG. 2S, at 206 the analysis application 118 analyzes the RRI data 104 to "identify “dips.” As explained in greater detail with reference to FIG. 4S, at 402 the analysis application 118 divides the RRI data 104 into segments (e.g., one-minute segments) and at 404 the analysis application 118 calculates the average RRI value for each segment. The analysis application 118 at 406 analyzes each point of the RRI data 104 and divides it by the average RRI value calculated for the segment in which the RRI data 104 point belongs. If the analysis application 118 determines at 408 that this ratio is less than a predefined threshold value, (e.g., 90%), at 410 the analysis application 118 stores or marks the interval index, RRI length, time at which the RRI length was found, and the ratio for further processing. It should be noted that 90% is merely one example, and other thresholds may additionally or alternately be determined, for example, 85%, 80%, 75%, 70%, 65%, or 60% as some other possibilities. In cases where additional thresholds are used, the analysis application 118 may provide indices for each. Regardless, RRI values for which the ratio is less than the predefined threshold value may be referred to as “dips.”

At 412, the analysis application 118 calculates time differences between the dips. The analysis application 118 analyzes these dips in chronological order and places them into groups at 414. The analysis application 118 may optionally perform filtering of the dips to remove standalone dips. Standalone dips may refer to dips flanked on both sides by an RRI ratio above the predefined threshold (e.g., above 90%). Such dips may be removed as they do not contribute to a greater trend of decreasing or increasing the RRI ratio. Regardless of whether the filtering is performed, analysis application 118 creates a group and places all dips in it until analysis application 118 identifies a pair of dips separated by more than a predefined time interval (e.g., ten seconds in an example, although greater or lesser time periods may be used). If so, the analysis application 118 creates a new group for the separate dip. The analysis application 118 analyzes and groups all of the “dips” in this manner. Using the determined groups, at 416 the analysis application 118 identifies the largest dip (i.e., the one with the smallest ratio) in each group and notes these as the biggest dips per group. The number of the biggest dips in the overall data file may be referred to as the RE RRDI (Respiratory-Related RR Interval drops).

At 418, the analysis application 118 divides the RE RRDI by the total time length of the file to determine the RE RRDI. The RE RRDI may refer to a measure of the overall events per unit of time, e.g., per hour in some examples. As mentioned above, one aspect of cleaning the RRI data 104 may include removing indexed data points for artifact data from the RRI data 104. When determining the RE RRDI, the analysis application 118 may detect when intervals have been removed if there is a skip in the index column (for example, for the indices [1, 2, 3, 5, 6], index 4 was skipped). The analysis application 118 may accordingly determine the amount of time elapsed between the pairs of data points flanking each skipped data point, sums these amounts of time, and subtracts the sum from the total length of the study. This difference may be referred to as the “corrected time.” The analysis application 118 may accordingly use the corrected time as the total time length in determining the overall RE RRDI. After 418, process 400 ends, and the flow return to process 200.

Generally, an increased RE RRDI measure may correlate to an increased risk of adverse cardiovascular outcomes, such as heart attack, heart failure, cardiac procedure, or cardiac death. Accordingly, the RE RRDI measure may be compared against a cutoff value to determine whether the patent whose data is being analyzed should be flagged for follow-up screening. This cutoff value may be scaled according to various factors, such as patient age, gender, weight, or other demographic or health risk information (e.g., smoker vs. non-smoker).

Referring back to FIG. 2S, at 208 the analysis application 118 number of desaturations that are greater than or equal to a predefined amount (e.g., greater than 3%). At 210 calculates the ODI, which refers to a measure of the number of events identified at 208 over time. For instance, the ODI may refer to an hourly index of the number of 3% events per hour. At 212, using the RRI data 104, the SaO2 data 108, and the information marked or stored above, the analysis application 118 generates the results 122. These results 122 may be provided to display panel 124.

FIG. 5S illustrates an example graphical illustration 500 of the results 122 of the process 200 for determining whether a patient has SDB. As shown in illustration 500, the RRI data 104 and the SaO2 data 108 are graphed over time with the same time scale.

More specifically, the RRI data 104 may be graphed to illustrate the raw data before processing (e.g., shown in blue). The RRI data 104 may also be illustrated with the calculated average (e.g., a minute average computed at operation 306). This average is shown in black. The dips computed at operation 410 may also be shown, indicated in the illustration as the red dots for dips to be considered and as the black asterisks for standalone dips that are filtered out.

Regarding the SaO2 data 108, the raw data may be displayed along with the relative maximums and minimums. For instance, indications of a first size and/or color (e.g., large and red triangles) may indicate maximums or minimums of 3% or more, indications of a second size and/or color (e.g., medium and purple triangles) may indicate maximums or minimums of 2% to 3%, and indications of a third size and/or color (e.g., small and blue triangles) may indicate maximums or minimums of 1% to 2%. Continuing with the example of triangular indications, upward-pointing triangles may refer to relative maximums, while downward pointing may triangles refer to relative minimums.

**How to generate RRI:**

-Open LabChart and load EDF

-Navigate to the HRV tab and select the EKG channel for analysis

-Create a tachogram by plotting the RRI values extracted by LabChart in another channel

-Visually inspect the data. Increase the retrigger delay if noise is creating noise with smaller RRI values and decrease the beat detection threshold if R waves are not being caught.

-After optimizing the accuracy of the tachogram, select upper and lower RRI limits between which the vast majority of actual, non-arrhythmic beats fall.

-Note the end time of the portion of the file, stopping before the EKG leads were removed.

-Export the file in a MATLAB file, saving the EKG, tachogram, SaO2, and HR files. Name the file with the lower and upper RRI limits, the end time in seconds, and the name of the subject analyzed.

-Analyze the exported file in MATLAB.

**Figures Legends**

FIG. 1S illustrates an example system for analyzing whether a patient has SDB.

FIG. 2S illustrates an example process for determining whether a patient has SDB according to the analysis of SaO2 data and analysis of RRI data.

FIG. 3S illustrates further details of the analysis of the SaO2 data to identify desaturation events.

FIG. 4S illustrates further details of the analysis of the RRI data to identify dips.

FIG. 5S illustrates an example graphical illustration of the results of the process for determining the RRI dips index and derived AHI based on raw ECG signal (A, B) that is processed to create RRI and O2 saturation dips (C).

FIG. 6S illustrates the ROC for respiratory-related RRDI (A) and HRAI (B) respectively. Panel C and D for total RRDI and GRAI respectively at four levels of AHI.

Figure 1S:


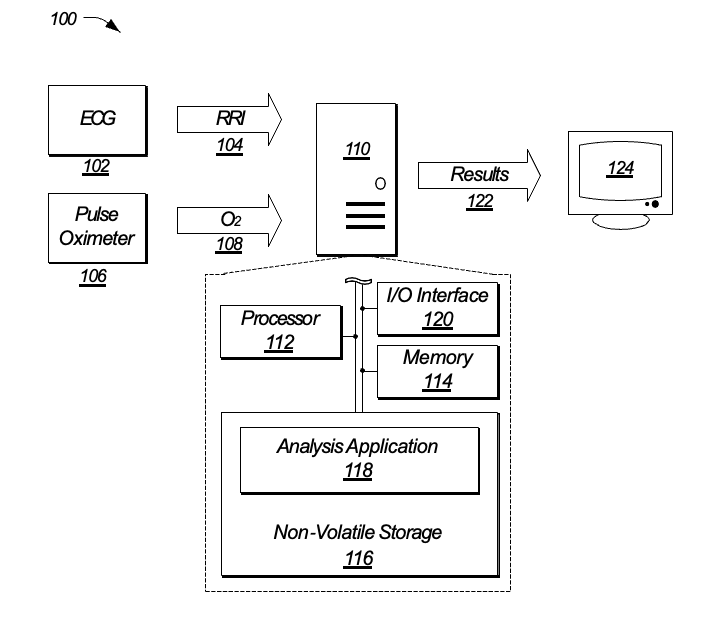


Figure 2S:


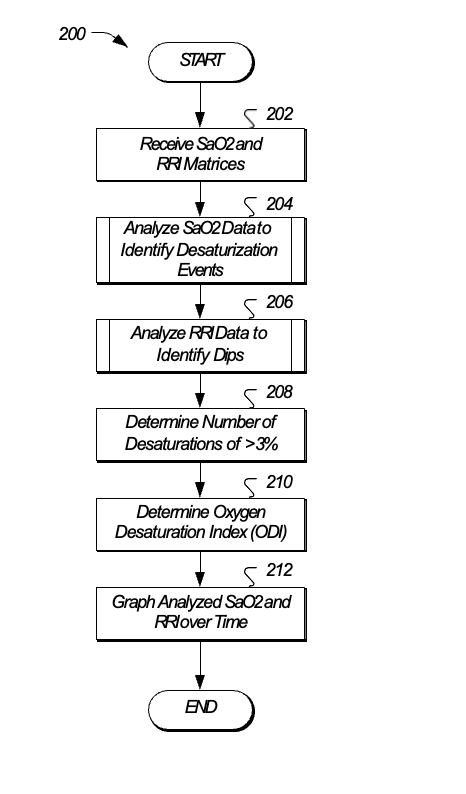


Figure 3S:


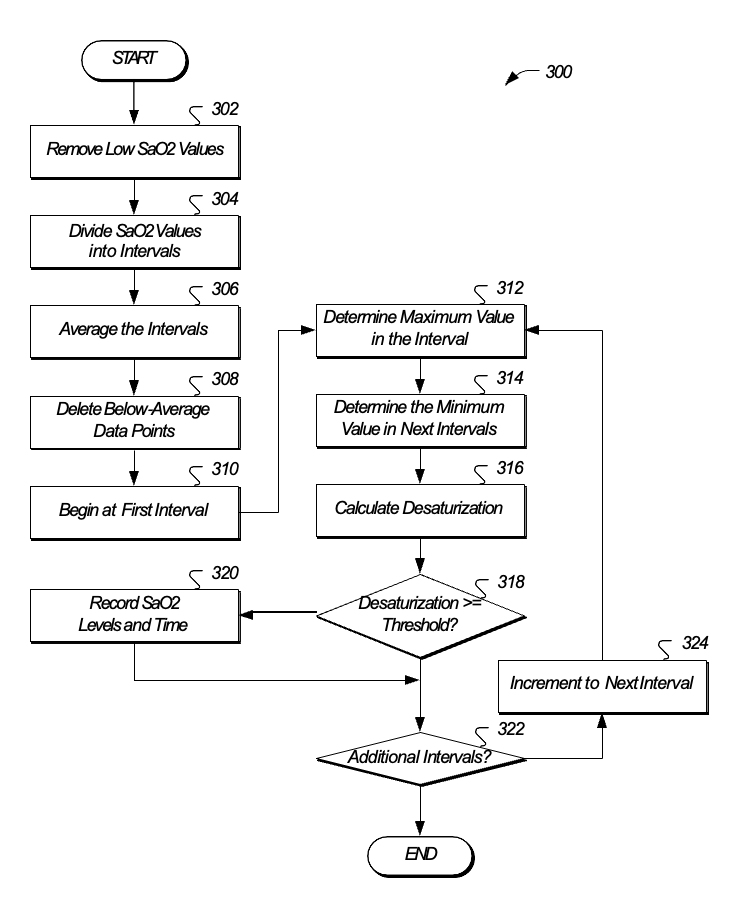


Figure 4S:


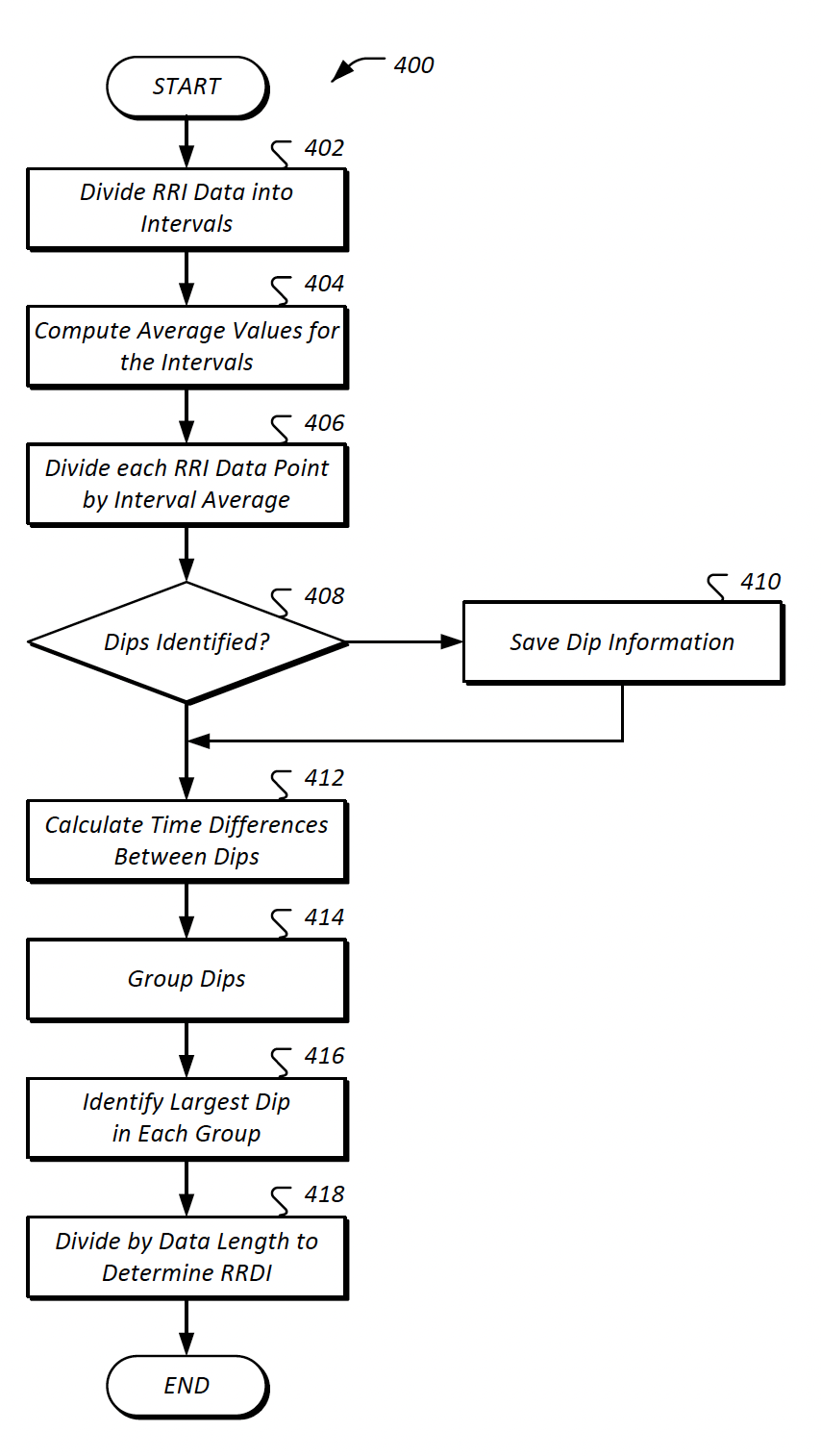


Figure 5S:


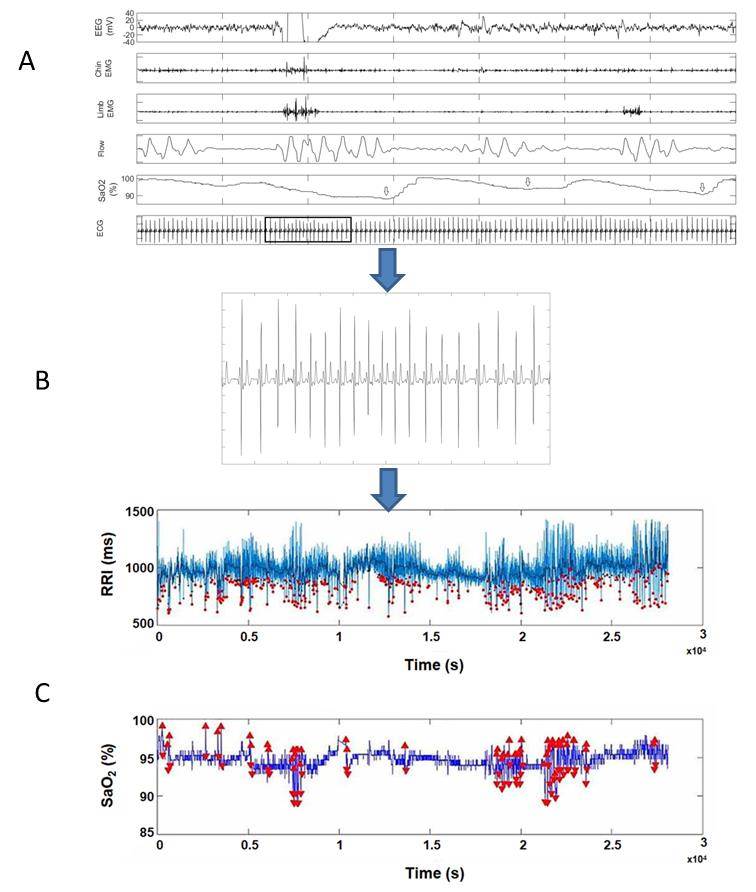


Figure 6S:

(A)

(B)

(C)

(D)

**Table 1S: Agreement Characteristics with RE RRDI >=5 for Validation Data Set**

a) Age < 55

|  | Total AHI Cut Off | | | |
| --- | --- | --- | --- | --- |
|  | AHI 5 | AHI 10 | AHI 15 | AHI 30 |
| Sensitivity, % | 61% | 78% | 82% | 83% |
| Specificity, % | 100% | 81% | 69% | 57% |
| PPV, % | 100% | 81% | 61% | 28% |
| NPV, % | 37% | 77% | 87% | 94% |
| LR + | 3.4504E9 | 4.0 | 2.7 | 1.9 |
| LR - | 0.4 | 0.3 | 0.3 | 0.3 |
| Agreement, % | 68% | 79% | 74% | 61% |
| Kappa | 0.37 | 0.58 | 0.48 | 0.22 |
| P-value | <0.0001 | <0.0001 | <0.0001 | <0.0001 |

b) age Age> 55

|  | Total AHI Cut Off | | | |
| --- | --- | --- | --- | --- |
|  | AHI 5 | AHI 10 | AHI 15 | AHI 30 |
| Sensitivity, % | 53% | 60% | 67% | 69% |
| Specificity, % | 100% | 92% | 84% | 59% |
| PPV, % | 100% | 96% | 88% | 38% |
| NPV, % | 17% | 40% | 59% | 84% |
| LR + | 3.2586E8 | 7.7 | 4.0 | 1.7 |
| LR - | 0.5 | 0.4 | 0.4 | 0.5 |
| Agreement, % | 57% | 67% | 73% | 62% |
| Kappa | 0.16 | 0.36 | 0.46 | 0.22 |
| P-value | <0.0001 | <0.0001 | <0.0001 | <0.0001 |

**Table 2S: Agreement Characteristics with RE HRAI >=5 for Validation Data Set**

a) Age < 55

|  | Total AHI Cut Off | | | |
| --- | --- | --- | --- | --- |
|  | AHI 5 | AHI 10 | AHI 15 | AHI 30 |
| Sensitivity, % | 70% | 92% | 98% | 96% |
| Specificity, % | 100% | 81% | 67% | 51% |
| PPV, % | 100% | 84% | 63% | 28% |
| NPV, % | 43% | 90% | 98% | 98% |
| LR + | 5.8709E9 | 4.7 | 3.0 | 1.9 |
| LR - | 0.3 | 0.1 | 0.03 | 0.08 |
| Agreement, % | 76% | 87% | 78% | 58% |
| Kappa | 0.47 | 0.73 | 0.58 | 0.24 |
| P-value | <0.0001 | <0.0001 | <0.0001 | <0.0001 |

b) Age > 55

|  | Total AHI Cut Off | | | |
| --- | --- | --- | --- | --- |
|  | AHI 5 | AHI 10 | AHI 15 | AHI 30 |
| Sensitivity, % | 77% | 86% | 91% | 97% |
| Specificity, % | 100% | 87% | 67% | 40% |
| PPV, % | 100% | 96% | 83% | 37% |
| NPV, % | 29% | 65% | 81% | 97% |
| LR + | 2.3636E8 | 6.7 | 2.8 | 1.6 |
| LR - | 0.00 | 0.2 | 0.1 | 0.07 |
| Agreement, % | 79% | 87% | 83% | 56% |
| Kappa | 0.36 | 0.66 | 0.60 | 0.25 |
| P-value | <0.0001 | <0.0001 | <0.0001 | <0.0001 |
